# Tuning electronic and optical properties of monolayer PdSe_2_ by introducing defects: first-principles calculations

**DOI:** 10.1038/s41598-020-60949-9

**Published:** 2020-03-04

**Authors:** X. W. Zhao, Z. Yang, J. T. Guo, G. C. Hu, W. W. Yue, X. B. Yuan, J. F. Ren

**Affiliations:** 1 School of Physics and Electronics, Shandong Normal University, Jinan, 250014 China; 2 Shandong Provincial Engineering and Technical Center of Light Manipulations & Institute of Materials and Clean Energy, Shandong Normal University, Jinan, 250014 China

**Keywords:** Condensed-matter physics, Electronics, photonics and device physics

## Abstract

Based on the density functional theory, the electronic and optical properties of pristine monolayer PdSe_2_ with Pd or Se vacancy-defect are investigated. Our results show that the Se defect is energetically more favorable than that of Pd defect. The band gap reduces, and some new midgap states appear after the Pd or Se defects are introduced. In terms of the optical properties, the prominent anisotropic characters are remained. The obvious new peaks of the dielectric constant appear after introducing defects. The light absorption in the visible energy range expands based on the appearance of the midgap states induced by the Pd or Se defects. The changes of the refractive index and reflectivity are similar with those of the dielectric constants and the light absorption. The energy loss spectrum of the PdSe_2_ with Pd or Se defects is obviously different, which can be used to identify different defects in PdSe_2_. These findings provide effective strategies to tune electronic and optical properties of monolayer PdSe_2_ by introducing defects.

## Introduction

In the last decades, the successful stripping of graphene greatly stimulates people’s interest in the study of two-dimensional (2D) materials^1–5^. However, the zero-band gap of graphene limits its applications in electronics, which leads to the emergence of other 2D materials beyond graphene, such as black phosphorous, boron nitride and transition metal di-chalcogenides (TMDCs)^6–11^. The common formula for TMDCs is MX_2_, where the M and X represent transition metals and chalcogen, respectively^12^. The favorable band-gap phenomenon together with the excellent chemical properties of TMDCs enable a wide range of application prospects in field-effect transistors, energy storage, catalysis, and so on^10,11^. Besides, several TMDCs have been widely studied both theoretically and experimentally based on their distinguished mechanics, electronics and optical properties, such like MoS_2_, MoSe_2_, and WS_2_^13–15^. Recently, another class of layered materials formed by noble metals (e.g. Pd and Pt) with S or Se atoms have been widely investigated owing to their unique atomic and electronic structures^16,17^.

Monolayer PdSe_2_, which is a special 2D material, possesses an uncommon pentagonal structure. Pd atoms coordinate with four Se atoms to form a square backbone network. Monolayer PdSe_2_ has been successfully exfoliated by Akinola D. Oyedele *et al*. for the first time, which provides exciting opportunities for the research of pentagonal 2D materials^18^. The air-stability and anisotropy of monolayer PdSe_2_ have been proved, moreover, few-layered PdSe_2_ behaves ambipolar semiconducting with high electron-apparent field-effect mobility. In addition, the remarkable electronic structures of PdSe_2_ are layer-dependent. The monolayer PdSe_2_ has an indirect band gap about 1.43 eV, while bulk PdSe_2_ has a band gap of 0.03 eV^18,19^. The promising thermoelectric performance of monolayer PdSe_2_ has been demonstrated through the density functional theory and semiclassical Boltzmann transport equation by Dan Qin *et al*. as well^20^. Overall, the discovery of pentagonal PdSe_2_ makes the emerging physics related to such low-symmetry structure possible. What’s more, monolayer PdSe_2_ can be a promising candidate in the applications of piezoelectrics, valleytronics, optoelectronic and spintronics.

In order to effectively optimize and utilize the excellent properties of 2D materials, various strategies to tune the optical and electronic properties of 2D materials have been adopted, such as the introduction of defects^21^, electric field and strain modulation^19,22^, atom doping and adsorption^23,24^, strain engineering^25^ etc. As we all know, defects can tune the electronic^26^, magnetic^27^, optical properties and enhance the electrochemical activity^19,28^. The existence of the point defects in semiconductors can efficiently trap free electrons, holes and localize excitons. When it recombines radiatively, the excitons can lead to light emission at energies lower than the band-to-band optical transition energy. On account of tighter localization of the electron wavefunction, the interactions between the defects and excitons become stronger in reduced dimensionalities materials^29^. On one hand, for monolayer PdSe_2_, it is highly desirable to explore the optical and electronic properties and develop simple and effective strategies to improve them. This will not only help to enhance the performance of device dependent on monolayer PdSe_2_ but also contribute to its applications in nanoelectronic devices. On the other hand, Lin *et al*. have reported that they have synthesized a novel 2D material monolayer Pd_2_Se_3_, which is a fusion of two defective PdSe_2_ layers due to the Se vacancies with a certain concentration^30,31^. They have systematically stated that the monolayer Pd_2_Se_3_ has excellent anisotropic electronic and optical properties and is a very promising candidate for photovoltaics. Take these things into account, we can infer that it is valuable to investigate the effects of the defects on the electronic and optical properties of PdSe_2_. In this work, we perform the first-principles calculations to investigate the electronic and optical properties of monolayer PdSe_2_ with Pd or Se vacancy defects, the Pd or Se defects are introduced by the random elimination of atoms. After introducing defects, obvious changes for electronic and optical properties of PdSe_2_ can be obtained.

## Results and Discussions

Firstly, the structural parameters of PdSe_2_ monolayer, PdSe_2_ with Pd or Se defects are calculated. The bond lengths and angles of vacancy-defected PdSe_2_ have little changes compared with the primitive cell, which means that there is only a slight distortion of the system, so the PdSe_2_ with a Pd or Se defect is stable. In order to further compare these two defects, the formation energies are obtained through the following equation ${E}_{form}={E}_{defect}-{E}_{pristine}\pm {\mu }_{n}$, where *E*_*defect*_ represents total energies of the relaxed PdSe_2_ with Pd or Se defect, *E*_*pristine*_ is the energy of the pristine PdSe_2_, *μ*_*n*_ is the chemical potential of the Pd or Se atom defect. The more positive the *E*_*form*_ is, the more difficult the defect to be formed. From our calculations, it can be obtained that the *E*_*form*_ of PdSe_2_ with Se defect is 1.38 eV, while for the PdSe_2_ with Pd defect, it is 1.97 eV, so Se defect is the more energetically favorable type. Recent work also has experimentally demonstrated the presence of Se defect in PdSe_2_^32^.

**Figure 1:**
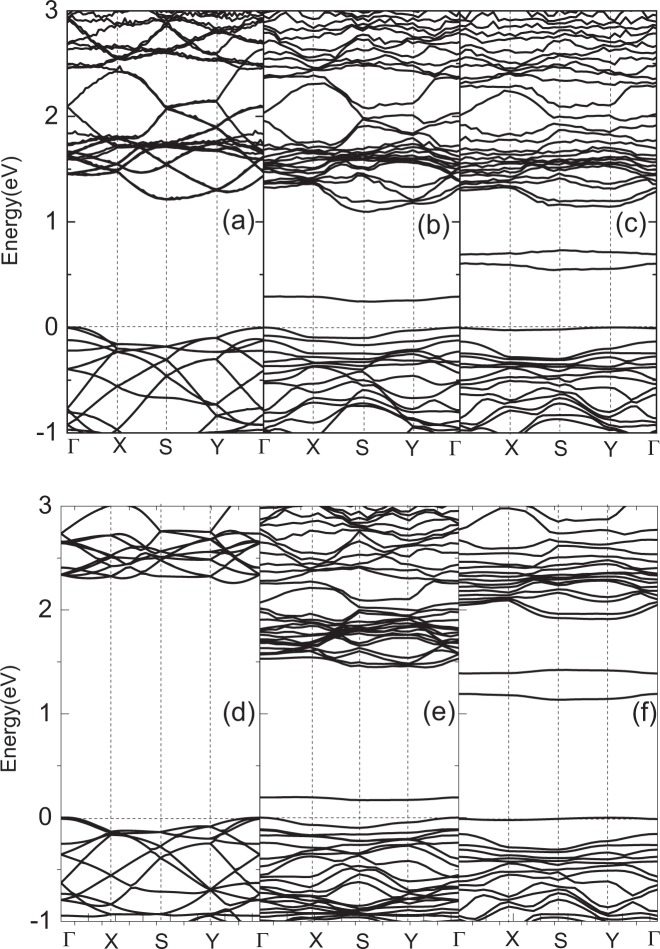
Band structures with PBE for pristine PdSe_2_ (**a**), PdSe_2_ with Pd defect (**b**) and Se defect (**c**), respectively. (**d–f**) Correspond to the cases with HSE06.

To further clarify the effects of defect on the electronic properties of monolayer PdSe_2_, the band structures along high symmetry k-points are depicted in Fig. 1. Figure 1(a–c) represent the band structures with PBE, Fig. 1(d–f) represent the band structures based on HSE06, respectively. By contrast, it can be found that the PBE underestimates the band gap, the HSE06 is more reasonable to calculate the electronic and optical properties, so the HSE06 are adopted in all the following calculations. As shown in Fig. 1(d), the pristine structure of monolayer PdSe_2_ has an indirect band gap about 2.25 eV, which is consistent with previous work^33^. However, after introducing Pd or Se defects, the band gaps are 1.45 and 1.91 eV, respectively. It can be observed from Fig. 1(e,f) that the presence of Pd or Se defect leads to some different new midgap states within the energy band gap, which can also be identified by the sharp peaks in the density of states. All the systems remain indirect band gap, and the PdSe_2_ with Pd defect still shows semiconductor characteristics. For the PdSe_2_ with Se defect, the band of midgap state goes through the Fermi level, the PdSe_2_ shows metal characteristics. It is also indicated that introducing defects is a potentially useful strategy to tune the band gap of 2D materials.

**Figure 2:**
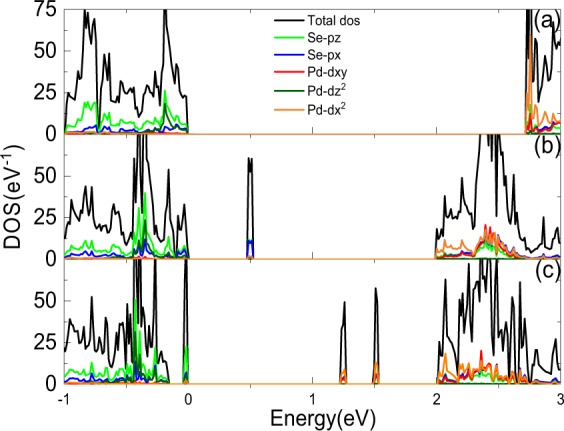
Density of states for different systems. (**a–c**) correspond to pristine PdSe_2_, PdSe_2_ with Pd or Se defect, respectively.

The total density of states (TDOS) and projected density of states (PDOS) for pristine PdSe_2_, PdSe_2_ with Pd or Se defect are shown in Fig. 2. For pristine PdSe_2_, it can be found that the dx_2_, dxy orbital of Pd atoms and the px, pz orbital of Se atoms mainly contribute to the states at conduction band edge, the dz^2^ orbital of Pd atoms and the px, pz orbital of Se atoms mainly contribute to the valence band edge. Besides, there is significant hybridization between Pd d and Se p states. While after introducing the defects, there are some changes, as shown in Fig. 2(b,c). It is obvious that the midgap states are mainly originated from the p^x^ and pz orbital of Se atoms in PdSe_2_ with Pd defect, while for PdSe_2_ with Se defect, the midgap states mainly originate from dx^2^ and dxy orbital. In general, the appearance of the peaks in the gapped region of the DOS are associated with the midgap states localized around the defects, which arises from the dangling bonds of Pd or Se due to their unsaturated charges, and their strength depends on the different missing atoms^34^. Thus one can identify the types of defect through the PDOS and then characterize the PdSe_2_ in order to get expected electronic properties.

**Figure 3:**
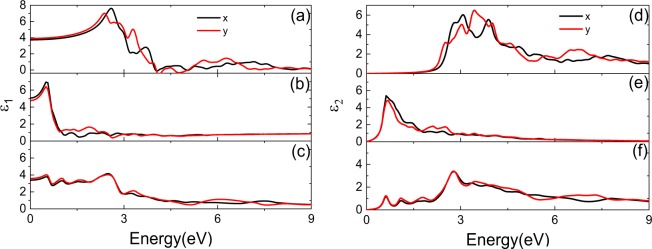
Dielectric constant of monolayer PdSe_2_. (**a–c**) Are the real part of dielectric function ɛ_1_ for pristine PdSe_2_, PdSe_2_ with Pd and Se defect, (**d–f**) Correspond to the imaginary parts ɛ_2_.

Optical properties of the materials are closely connected to its electronic properties. And it is evident from the previous calculations that the defects can alter the electronic properties of monolayer PdSe_2_, the change of electronic properties is expected to modify the optical properties. Figure 3(a–c) represent the real parts of the dielectric constant *ε*_1_ for pristine PdSe_2,_ PdSe_2_ with Pd or Se defect, (d)–(f) represent the corresponding imaginary parts *ε*_2_, respectively. From Fig. 3(a–c), it can be found that the maximum value of *ε*_1_ in *x* direction for pristine PdSe_2_ is about 7.62, however, it reduces to 6.94 and 4.15 for PdSe_2_ with Pd or Se defect, respectively. The maximum values are 7.06, 6.41 and 4.02 for pristine PdSe_2_, PdSe_2_ with Pd or Se defect in *y* direction of *ε*_1_, which is different from those in *x* direction. After introducing defect, the maximum value of *ε*_2_ also decreases from 6.03 for pristine PdSe_2_ to 5.39 and 3.36 for PdSe_2_ with Pd or Se defect in *x* direction, respectively. It can be found that the change of *ε*_2_ is different as well both in *x* and *y* directions, which means that the anisotropy of the optical properties of PdSe_2_ remains unchanged. Furthermore, the peaks in the low energy range of dielectric constant correspond to the peaks in the DOS, which mainly due to the new midgap states originated from the defects.

**Figure 4:**
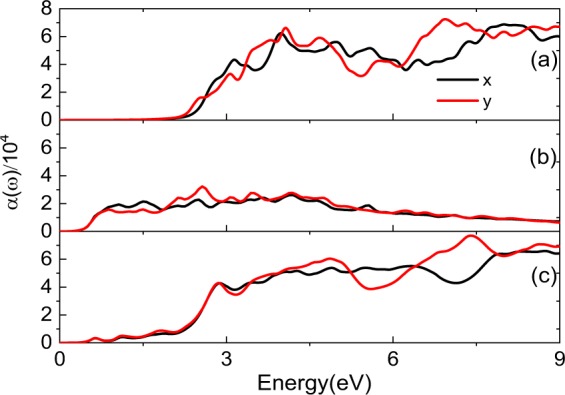
Absorption coefficient for pristine PdSe_2_ (**a**), PdSe_2_ with Pd (**b**) and Se defect (**c**), respectively.

The absorption coefficients *α(ω)* for pristine PdSe_2_, PdSe_2_ with Pd or Se defect have been depicted to reveal the light absorption properties of PdSe_2_, which are shown in Fig. 4. The optical absorption spectrum is closely related to the imaginary parts of the dielectric constant. It can be observed that there is no absorption within the energy range of 0 to 2.20 eV for pristine PdSe_2_ as shown in Fig. 4(a), which is consistent with the band gap structure in Fig. 1(d). According to the suitable band gap, monolayer PdSe_2_ is expected to be a promising candidate for light absorption. Monolayer PdSe_2_ exhibits good optical absorption in the visible regions (1.64–3.19 eV) as is shown in Fig. 4(a). After introducing the Pd or Se defect, the absorption is optimized obviously. The optical absorption starts from 0.44 and 0.53 eV for the PdSe_2_ with Pd or Se defect, which are shown in Fig. 4(b,c). That is to say, the optical absorption in low energy region widens, especially in the visible regions. That is benefit from the existence of the Pd or Se defect in PdSe_2_, they can create some midgap states, so more new optical transitions can be activated comparing with the case of pristine PdSe_2_. In addition, it can be found that the absorption coefficients are different in *x* and *y* directions.

**Figure 5:**
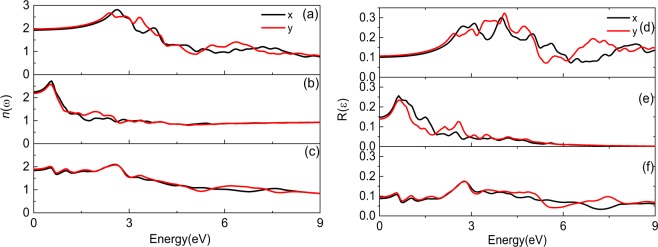
Refractive index and reflectivity of PdSe_2_ systems. (**a–c**) Correspond to refractive index, (**d–f**) correspond to reflectivity for pristine PdSe_2_, PdSe_2_ with Pd or Se defect, respectively.

Figure 5 shows the refractive index *n*(ω) and reflectivity *R*(ω) of PdSe_2_ systems. The maximum value of *n*(ω) for pristine PdSe_2_ is about 2.82. There is noticeable change for the maximum *n*(ω) of the PdSe_2_ with Pd or Se defect compared with that of pristine PdSe_2_, it decreases to 2.72 and 2.01. And the new peak of *n*(ω) appears in low energy region obviously as shown in Fig. 5(b,c), which is consistent with the dielectric constant. The *R*(ω) of PdSe_2_ systems are shown in Fig. 5(d–f) respectively. The maximum *R*(ω) for the pristine PdSe_2_ and PdSe_2_ with Pd or Se defects are 0.32, 0.25 and 0.17, respectively. It is obvious that the maximum *R*(ω) decreases compared with that in pristine PdSe_2_, and there are new peaks in low energy region after introducing Pd and Se defects. All the new peaks in low energy region are related to the new midgap states.

**Figure 6:**
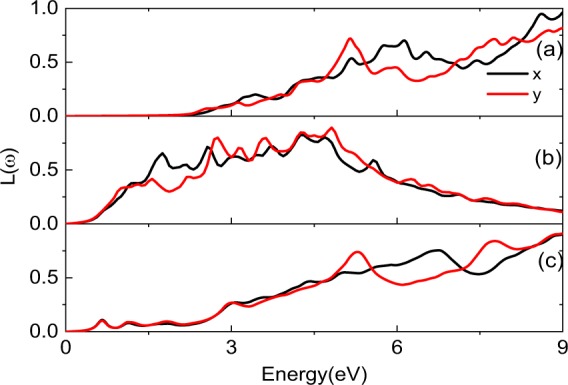
Electron energy loss spectrum for pristine PdSe_2_ (**a**), PdSe_2_ with Pd (**b**) or Se defect (**c**), respectively.

The electron energy loss spectrum *L(ω)* have also been depicted for pristine PdSe_2_, PdSe_2_ with Pd or Se defect, which are shown in Fig. 6(a–c), respectively. By contrast, it can be found that obvious differences occurred due to the introduction of the defects. The *L(ω)* starts from 2.31, 0.31 and 0.45 eV for pristine PdSe_2_, PdSe_2_ with Pd or Se defect, respectively. The first sharp peak of *L(ω)* is observed at 5.13, 1.75 and 0.68 eV. Besides, the curves of pristine PdSe_2_ and PdSe_2_ with Se defect have a tendency of ascending first and descending in succession then ascending. While the curve of PdSe_2_ with Pd defect has a tendency of ascending first and descending in succession, and it has relatively dense peaks. The different *L(ω)* can be used to identify different defects.

## Conclusions

In this work, the electronic and optical properties of pristine PdSe_2_, PdSe_2_ with Pd or Se defect are studied through the first-principles calculations based on the density functional theory. The result of the formation energies indicates that Se defect is more energetically favorable compared with that of Pd defect. By calculating the band structures, it is found that the band gap reduces due to the appearing of the new midgap states after introducing Pd or Se defect. The midgap states are originated from the dangling bonds of Pd or Se due to their unsaturated charges. In terms of the optical properties, the dielectric constant and absorption spectrum, refractive index, reflectivity and electron energy loss spectrum have been analyzed. The obvious peaks of the dielectric constant and the absorption spectrum in low energy region can be observed due to the appearance of the new midgap states induced by the Pd or Se defect. And the prominent anisotropic characters are remained. The light absorption area in the visible regions widens after introducing Pd or Se defect compared with that of pristine PdSe_2_. The new peaks benefit from the appearance of the midgap states which activate more new optical transitions in the optical spectrum. For the refractive index and reflectivity, the similar changes have been taken place. Furthermore, the difference of the electron energy loss spectrum can be used to identify different defects. All these findings provide effective strategies to tune electronic and optical properties of monolayer PdSe_2_, and provide possibilities for monolayer PdSe_2_ in the applications of optoelectronics.

### Theoretical model and computational details

**Figure 7:**
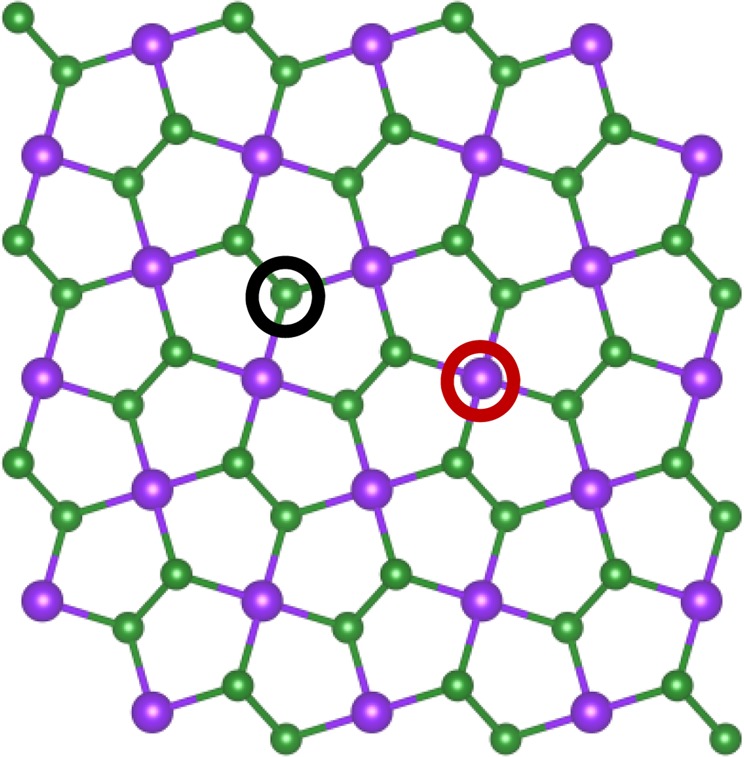
Top view of PdSe_2_ monolayer. Green and purple spheres represent Se and Pd atoms, red and black circle represent Pd and Se vacancy-defect positions, respectively.

All our theoretical calculations are performed through VASP (Vienna ab-initio Simulation Package)^35,36^, PAW pseudopotential is used to describe the interaction between ions and electrons^37,38^. In terms of the energy exchange correlation energy function, the generalized gradient approximation (GGA) in the form of Perdew-Burke-Ernzerhof (PBE) is used^39^. The electronics and optical properties are calculated by the hybrid functional based on the Heyd-Scuseria-Ernzerhof (HSE06) exchange-correlation functional^40^. The cut-off energy is 400 eV in the process of the structural optimizations and calculations. The 11 × 11 × 1 Monkhorst-Pack grid is chosen when calculating the integral in Brillouin zone. Energy convergence is set to less than 10^−4^ eV and the convergence accuracy of the nuclear motion is set to less than 0.01 eV/Å. A 3 × 3 × 1 supercell (54 atoms) of PdSe_2_ is constructed, as shown in Fig. 7. It is worth noting that the residual strain may exist due to the limit of periodic boundary condition no matter how bigger the supercell is. In order to avoid the interlayer interference, the thickness of the vacuum layer is set to 20 Å.

The optical properties are general evaluated by the dielectric function which are the sum of real and imaginary parts, $\varepsilon (\omega )={\varepsilon }_{1}(\omega )+i{\varepsilon }_{2}(\omega )$. The imaginary part is calculated by the summation of empty band states using the following equation^41^,

1 $${\varepsilon }_{2}(\omega )=\frac{4{\pi }^{2}{e}^{2}}{\Omega }\mathop{\mathrm{lim}}\limits_{q\to 0}\frac{1}{{q}^{2}}\sum _{c,v,k}2{\omega }_{k}\delta (\,{\in }_{ck}-{\in }_{vk}-\omega )\times \langle {u}_{ck+{e}_{\alpha }q}|{u}_{vk}\rangle {\langle {u}_{ck+{e}_{\beta }q}|{u}_{vk}\rangle }^{\ast }$$

where *Ω* represents the volume, *v* and *c* correspond to the valence and the conduction band respectively, *α* and *β* indicate the Cartesian components, *e*_*α*_ and *e*_*β*_ are the unit vectors, ${{\in }}_{ck}$ and ${{\in }}_{vk}$ refer to the energy of conduction and valence band respectively, *u*_*ck*_ is the cell periodic part of the orbitals at the k-point. The real part of dielectric constant is calculated by the Kramers-Kronig relation^42^

2 $${\varepsilon }_{1}(\omega )=1+\frac{2}{\pi }P{\int }_{0}^{\infty }\frac{{\varepsilon }_{2}^{\alpha \beta }(w{\prime} )w{\prime} }{{w{\prime} }^{2}-{w}^{2}+i\eta }d\omega {\prime} ,$$

with P being the principle value. According to the values of real and imaginary part of the dielectric constant, the optical absorption coefficient *α(ω)*, the refractive index *n(ω)*, the reflectivity *R(ω)*, and the electron energy loss spectroscopy *L(ω)* can be given by^43^,

3 $$\alpha (\omega )=\frac{\sqrt{2}\omega }{c}{\{{[{\varepsilon }_{1}^{2}(\omega )+{\varepsilon }_{2}^{2}(\omega )]}^{\frac{1}{2}}-{\varepsilon }_{1}(\omega )\}}^{\frac{1}{2}},$$

4 $$n(\omega )=\frac{1}{\sqrt{2}}{\{{[{\varepsilon }_{1}^{2}(\omega )+{\varepsilon }_{2}^{2}(\omega )]}^{\frac{1}{2}}+{\varepsilon }_{1}(\omega )\}}^{\frac{1}{2}},$$

5 $$R(\omega )={|\frac{\sqrt{{\varepsilon }_{1}(\omega )+i{\varepsilon }_{2}(\omega )}-1}{\sqrt{{\varepsilon }_{1}(\omega )+i{\varepsilon }_{2}(\omega )}+1}|}^{2},$$

6 $$L(\omega )=\frac{{\varepsilon }_{2}(\omega )}{{\varepsilon }_{1}^{2}(\omega )+{\varepsilon }_{2}^{2}(\omega )}.$$
